# Low‐level human memory T and B cells recognising avian influenza hemagglutinins are poorly responsive to existing seasonal influenza vaccines

**DOI:** 10.1002/cti2.70067

**Published:** 2025-12-10

**Authors:** Christopher A Gonelli, Marios Koutsakos, Robyn Esterbauer, Ming ZM Zheng, Yee‐Chen Liu, Amanda Kyaw Zin, Lara SU Schwab, Aeron C Hurt, Stephen J Kent, Jennifer A Juno, Adam K Wheatley

**Affiliations:** ^1^ Department of Microbiology and Immunology Peter Doherty Institute for Infection and Immunity, The University of Melbourne Melbourne VIC Australia; ^2^ WHO Collaborating Centre for Reference and Research on Influenza Peter Doherty Institute for Infection and Immunity Melbourne VIC Australia

**Keywords:** avian influenza, B‐cell responses, CD4 T‐cell responses, cross‐reactive antibodies, influenza vaccination

## Abstract

**Objectives:**

Immunisation remains the most cost‐effective mechanism to combat influenza infection and is widely employed against seasonal influenza viruses. Zoonotic transmission of avian influenza A viruses represents a significant threat to human health given the lack of population‐level immunity. Therefore, there is a need to better understand pre‐existing cross‐reactive human immunity against avian influenza strains, as highlighted by the recent global spread of avian H5Nx clade 2.3.4.4b variants.

**Methods:**

Here, we quantified the frequencies and specificities of B and T cells recognising avian hemagglutinin (HA) within unexposed adults and characterised the ability of seasonal immunisation to boost cross‐reactive immune responses to H5Nx strains, including from clade 2.3.4.4b.

**Results:**

Low but detectable serum antibody titres against H5 and H7 avian influenza HA were observed in donors. The frequency of memory B cells with cross‐reactive recognition of H5 and H7 HA was below 0.13% and two‐ to five‐fold lower than populations of seasonal HA‐specific B cells. Boosting of B‐cell responses against clade 2.3.4.4b H5Nx HA following seasonal immunisation was sporadic with only three of 19 individuals showing an increased population of probe‐positive cells. Cross‐reactive B cells generally expressed immunoglobulins drawn from variable heavy chain genes associated with HA stem recognition. CD4^+^ T‐cell responses towards H5 HA were weakly boosted with little increase in circulating T follicular helper cell populations.

**Conclusion:**

These findings highlight the need for avian influenza‐specific vaccine products to bolster immunity in human populations. Such vaccines could aid pre‐pandemic preparedness by expanding baseline frequencies of avian influenza‐specific memory lymphocytes.

## Graphical abstract

In this study, we found low but detectable antibody responses against H5 and H7 avian influenza hemagglutinin in unexposed human cohorts. B‐ and T‐cell responses against these avian‐origin hemagglutinins were poorly boosted by seasonal influenza vaccination.

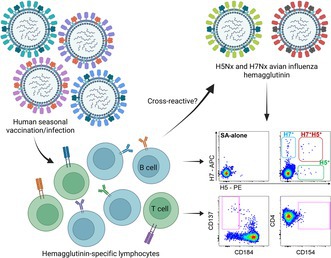

## Introduction

Zoonotic transmission of avian influenza A viruses, such as H5Nx and H7Nx, is often associated with extremely high pathogenicity and case fatality rates in humans.^1^, ^2^, ^3^, ^4^, ^5^ Due to a lack of population‐level immunity, cross‐over from avian reservoirs represents a pressing and emergent threat to human health, with any global pandemic having the potential to cause high mortality and massive social upheaval. While a variety of monovalent vaccines targeting H5Nx or H7Nx influenza have been produced and are immunogenic in humans,^6^, ^7^, ^8^, ^9^, ^10^ the unpredictable location and timing of any emergent pandemic makes antigenic mismatch between existing vaccines and/or vaccine seed stocks highly likely. There is therefore a need to better understand pre‐existing human immunity against avian influenza strains, particularly in light of the recent global spread of avian H5Nx 2.3.4.4b variants.^11^

The existence within unexposed individuals of serum antibodies able to bind and/or neutralise the hemagglutinin (HA) of avian influenza strains has been widely reported. H5 and/or H7 reactivity is observed in both pooled intravenous immunoglobulin (IVIG) preparations^12^, ^13^ and in serum samples from human cohorts,^14^, ^15^, ^16^ although reported serological concentrations are generally very low. In addition, monoclonal antibodies (mAbs) binding H5 or H7 isolates are readily isolated from subjects not directly exposed to avian influenza by immunisation or infection^17^, ^18^, ^19^ and can protect against H5Nx or H7Nx challenge in mice.^17^, ^18^, ^20^ Notably, immune exposure to antigenically divergent HA drives the preferential expansion of highly cross‐reactive antibody and memory B‐cell populations,^21^, ^22^, ^23^, ^24^ including those expressing rare mAbs able to neutralise and/or protect against both Group 1 and Group 2 influenza.^25^, ^26^ Thus, the human immune system appears highly capable of targeting conserved epitopes shared by seasonal and avian influenza strains.

Existing influenza‐specific cellular responses that are H5Nx virus cross‐reactive are also present within populations of unexposed individuals, as evidenced by CD4^+^ (and CD8^+^) memory T‐cell reactivity predominantly recognising internal proteins of avian viruses.^27^, ^28^, ^29^, ^30^, ^31^ This cross‐reactive memory is likely the product of epitope conservation between seasonal and avian viruses,^32^ and can be expanded following inactivated H5N1 virus immunisation.^33^ Studies suggest that the immunogenicity of HA‐based vaccines in humans is determined, in part, by levels of pre‐existing HA‐specific CD4^+^ memory T cells.^34^, ^35^ In populations with low baseline H5‐ or H7‐reactive T‐cell pools, vaccine immunogenicity may be improved by prior CD4^+^ T‐cell priming^33^ or by covalent coupling of novel HA antigens to seasonal HA proteins.^36^

Most recently, novel H5 viruses from clade 2.3.4.4b have spread globally through wild bird populations in four continents^37^ and outbreaks have been reported within domestic cattle,^38^, ^39^, ^40^ sea mammals^41^, ^42^ and mustelids.^43^ Disease course within mammals varies in severity, with a mild disease largely confined to mammary tissues reported in cattle, while infection in cats^44^ and experimentally infected naïve ferrets^45^ and non‐human primates^46^ can be highly pathogenic causing major lung pathology and/or death. To date, zoonotic infections in humans have been nearly universally mild, likely reflecting a degree of cross‐protective immunity within the population seeded by seasonal exposure.

Here, we sought to quantify the frequencies and specificities of B cells recognising avian HA within adults, and to determine the extent to which seasonal immunisation can boost cross‐reactive immune responses to H5, including lineages, such as 2.3.4.4b. We find memory B cells recognising HA from avian H5Nx and H7Nx influenza strains are widely prevalent in healthy unexposed Australian adults and utilise stereotypic immunoglobulin sequences previously shown to be able to protect in animal models. Vaccination with seasonal inactivated influenza vaccine drives a modest, transient expansion of B cells and CD4^+^ T cells recognising avian influenza strains. Our results suggest that while population‐level immunity to H5Nx 2.3.4.4b in the form of antibody and memory lymphocyte populations is widespread, targeted vaccine strategies against H5Nx will be required to markedly bolster immunity to emerging avian influenza threats.

## Results

### Antibodies and B cells binding hemagglutinin from H5 and H7 avian influenza strains are widely prevalent in unexposed adults

**Figure 1:**
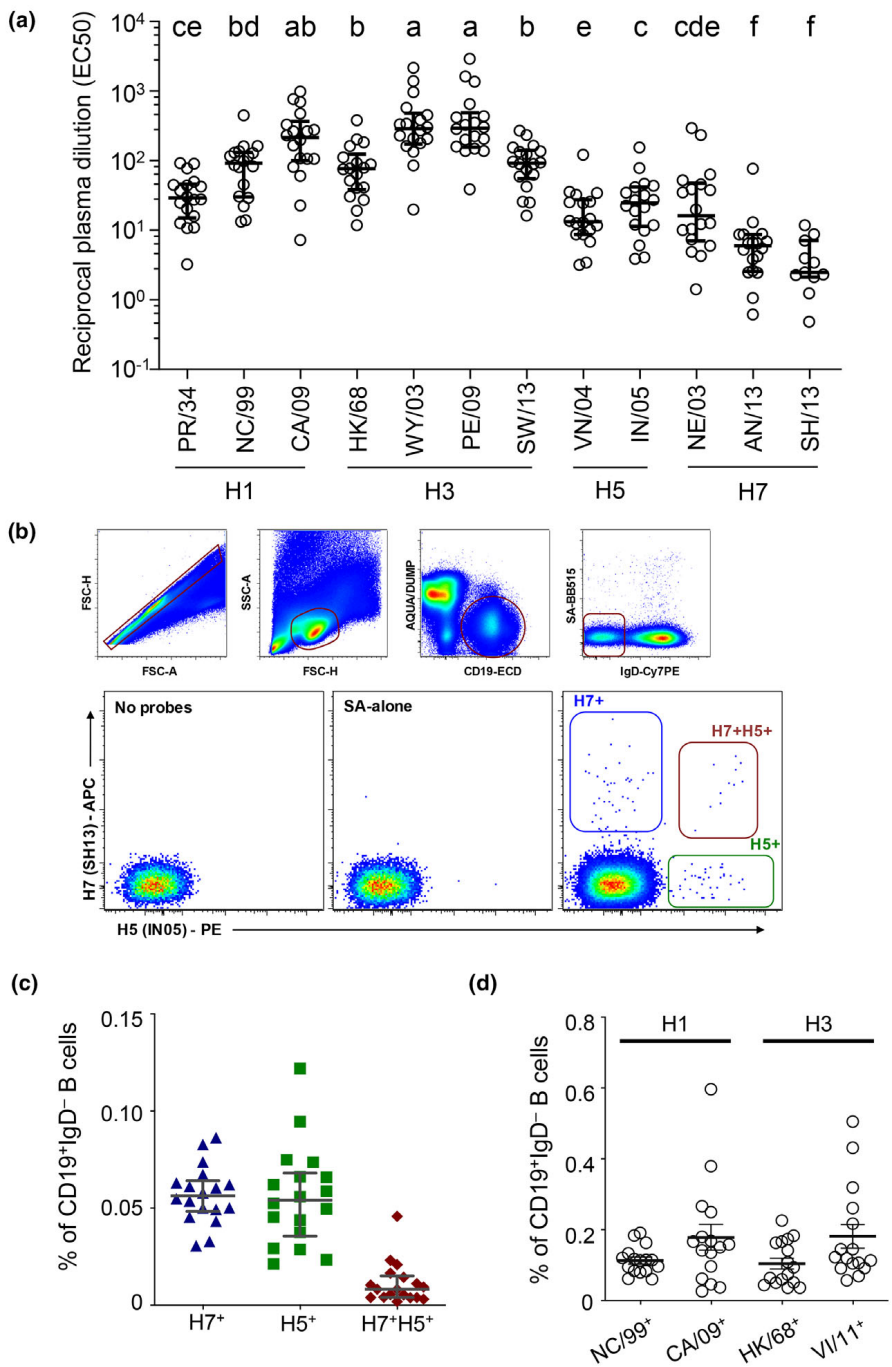
Serum antibody and memory B cells recognising avian influenza strains are widely prevalent. **(a)** Plasma samples from healthy volunteers (*N* = 18) were screened by enzyme‐linked immunosorbent assay (ELISA) for reactivity against hemagglutinin (HA) from diverse influenza A strains. Reciprocal plasma dilutions yielding half‐maximal binding (EC50) for each antigen are shown. Mixed‐effects ANOVA analysis, with the Geisser–Greenhouse correction, was used on matched samples followed by Tukey's multiple comparisons test on all pairs of antigens. The post‐test results are presented as compact letter display above each column, with columns that share a letter not being statistically different from each other (*P* ≥ 0.05). **(b)** Staining of cryopreserved PBMCs allows identification of CD19^+^ IgD^−^ B cells not binding a streptavidin‐BB515 decoy. Co‐staining with recombinant HA probes derived from H7N9 A/Shanghai/01/2013 (SH13) and H5N1 A/Indonesia/5/2005 (IN05) delineates single‐ and cross‐reactive memory B‐cell populations. **(c)** Frequencies of H5^+^, H7^+^ or H7^+^H5^+^ memory B cells in healthy volunteers (*N* = 18). **(d)** Frequencies of memory B cells binding seasonal H1N1 (NC99 and CA09) and H3N2 (HK68 and VI11) influenza strains were measured in healthy volunteers (*N* = 16). Lines indicate median and IQR. PR/34, A/Puerto Rico/8/1934(H1N1); NC/99, A/New Caledonia/20/1999(H1N1); CA/09, A/California/04/2009(H1N1); HK/68, A/Hong Kong/1/1968(H3N2); WY/03, A/Wyoming/3/2003(H3N2); PE/09, A/Perth/16/2009(H3N2); SW/13, A/Switzerland/9715293/2013(H3N2); VN/04, A/Vietnam/1203/2004(H5N1); IN/05, A/Indonesia/5/2005(H5N1); NE/03, A/Netherlands/219/2003(H7N7); AN/13, A/Anhui/01/2013(H7N9); SH/13, A/Shanghai/01/2013(H7N9); VI/11, A/Victoria/361/2011(H3N2).

We initially sought to determine the prevalence and magnitude of antibody titres against avian influenza strain‐origin HAs at baseline within a cohort of healthy Australian adults with no known exposure (*N* = 18). Consistent with previous reports,^14^, ^15^, ^16^ we observed low but detectable serum antibody titres reactive against H5 and H7 avian influenza strains (Figure 1a). The H5 and H7 strain titres were significantly lower (*P* ≤ 0.05 using mixed‐effects ANOVA analysis with the Geisser–Greenhouse and Tukey's multiple comparisons corrections) than those against recently circulating H1N1 (A/California/04/2009) and H3N2 (A/Wyoming/3/2003, A/Perth/16/2009 and A/Switzerland/9715293/2013) influenza strains, with between 5‐ and 118‐fold lower median titres observed. Memory B cells recognising HA from historical H5 [A/Indonesia/05/2005(H5N1)] and H7 [A/Shanghai/02/2013(H7N9)] avian influenza strains were quantified by flow cytometry using recombinant HA probes.^47^ Distinct H5^+^ and H7^+^ memory B‐cell populations were detected in all individuals tested (Figure 1b), with median respective frequencies of 0.054% (range 0.021–0.122) and 0.056% (range 0.031–0.086) of total class‐switched (IgD^−^) B cells (Figure 1c). Cross‐reactive memory B cells binding both H5 and H7 probes were comparatively infrequent (0.008%; range 0.002–0.046); however, strongly, dual‐probe staining populations were readily discernible in several subjects screened (Figure 1b and c). As a point of reference, memory B cells specific for seasonally epidemic H1N1 (A/New Caledonia/20/1999 and A/California/04/2009) and H3N2 (A/Hong Kong/1/1968 and A/Victoria/361/2011) influenza strains were commonly observed at frequencies two to five times greater within the same individuals (Figure 1d) (*N* = 16 matched subjects). Memory B cells specific for avian HA were phenotypically comparable to the parental memory B‐cell population, displaying a similar distribution of surface immunoglobulin usage (Supplementary figure 1–3a) and a predominantly resting memory phenotype (CD27^+^CD21^−^) (Supplementary figure 1–3b). There was a weak, but not significant, association between subject age and the frequency of cross‐reactive memory B cells, an observation that requires clarification in larger cohorts but is consistent with driving cross‐reactive responses from seasonal flu infections and/or vaccinations (Supplementary figure 1–3c).

### Recovered immunoglobulins recognise HA from diverse influenza subtypes

**Figure 2:**
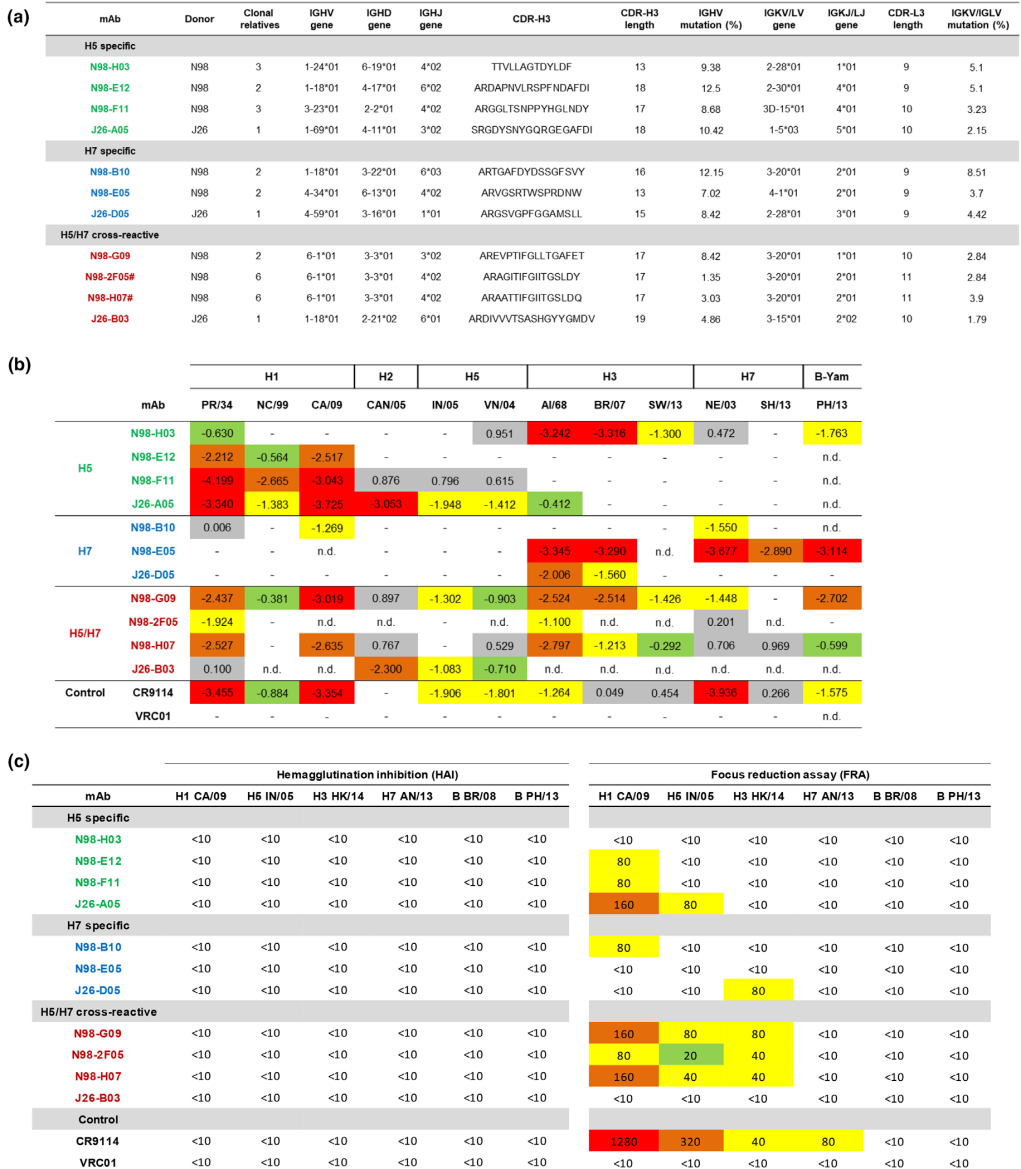
Characteristics of monoclonal antibodies derived from avian hemagglutinin (HA)‐specific B cells. **(a)** Gene usage, CDR‐H3 sequence and mutation loads for 14 monoclonal antibodies derived from sorted H5^+^, H7^+^ or H7^+^H5^+^ memory B cells from two healthy volunteers. **(b)** Binding activity of each monoclonal antibody to a panel of recombinant HA proteins derived from influenza A and influenza B strains. Values denote binding log_10_(EC50) determined by ELISA. HA and mAb combinations not tested are indicated by ‘n.d.’ **(c)** HA target receptor binding inhibition and neutralisation activity determined by hemagglutination inhibition (HAI) and focus reduction assay (FRA), respectively, against a panel of influenza A and B viruses. PR/34, A/Puerto Rico/8/1934(H1N1); NC/99, A/New Caledonia/20/1999(H1N1); CA/09, A/California/04/2009(H1N1); CAN/05, A/Canada/720/2005(H2N2); AI/68, A/Aichi/2/1968(H3N2); HK/14, A/Hong Kong/4801/2014(H3N2); BR/07, A/Brisbane/10/2007(H3N2); SW/13, A/Switzerland/9715293/2013(H3N2); VN/04, A/Vietnam/1203/2004(H5N1); IN/05, A/Indonesia/5/2005(H5N1); NE/03, A/Netherlands/219/2003(H7N7); AN/13, A/Anhui/01/2013(H7N9); SH/13, A/tree sparrow/Shanghai/01/2013(H7N9); BR/08, B/Brisbane/60/2008; PH/13, B/Phuket/3073/2013.

Single memory B cells binding H5, H7 or both HA probes were sorted from two individuals with the most robust responses and immunoglobulin gene sequences were recovered as previously described.^47^, ^48^ A small panel of monoclonal antibodies was expressed (Figure 2a) and binding to HA from diverse influenza strains was confirmed by ELISA using recombinant HA proteins (Figure 2b). Consistent with reports of broadly cross‐reactive human antibodies, mAbs derived from H5‐specific B cells primarily bound influenza A viruses from Group 1 (H1, H2 and H5), while those from H7‐specific B cells bound Group 2 (H3 and H7). Antibodies from B cells with H5/H7 cross‐binding activity bound more broadly and were generally drawn from IGHV6‐1 and IGHV1‐18 convergent classes as previously described.^26^, ^49^ The ability of the mAbs to inhibit HA target receptor binding and neutralise virus activity was assessed using hemagglutination inhibition (HAI) and focus reduction assays (FRA), respectively, against a panel of influenza A and B viruses (Figure 2c). None of the isolated mAbs showed HAI activity against H1, H5, H3 or H7 viruses nor influenza B viruses. However, most of the mAbs showed FRA activity against one or more HA subtypes, particularly for H5 among the H5/H7 cross‐reactive mAbs. As HAI exclusively measures antibodies targeting the HA head domain while FRA activity measures antibodies that inhibit virus spread (binding, fusion and release), this suggests that, as expected, mAbs are specific for the stem region of HA. This is consistent with the positive control stem‐binding mAb, CR9114, showing no HAI activity while having broad FRA activity against the influenza A viruses.^50^ Overall, pre‐existing neutralising antibodies binding the HA stem domain are prevalent among unexposed individuals and can exhibit neutralising and potentially protective activity against avian influenza strains.

### Responsiveness of memory B cells binding avian HA to vaccination or infection with seasonal influenza viruses

**Figure 3:**
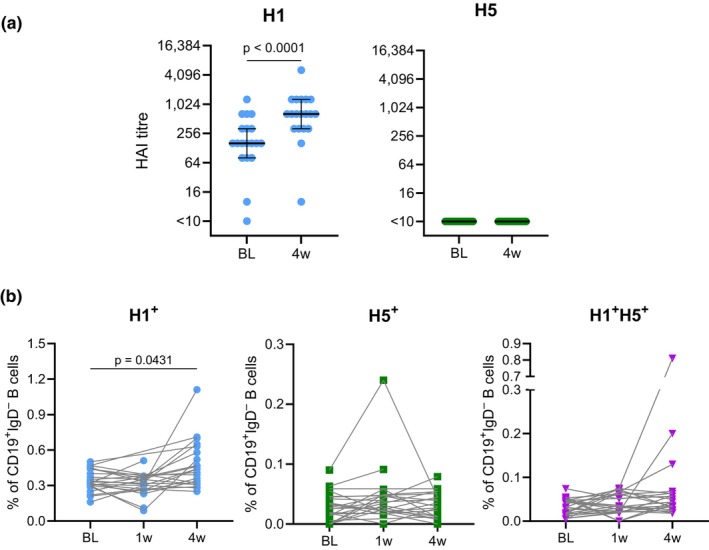
Cross‐reactive serum neutralising activity not induced by seasonal vaccination but sporadic induction of cross‐reactive H5 memory B cells. **(a)** Plasma samples from healthy volunteers (*N* = 19) were assayed for hemagglutination inhibition (HAI) against the vaccine matched H1N1 strain (A/Michigan/45/2015 pdm09) and clade 2.3.4.4b H5N1 strain (A/Fujian‐Sanyuan/21099/2017). The HAI assay employed virus particles to measure H1 responses whereas H5 HA‐ferritin nanoparticles were used as a surrogate for virus to assess H5 responses. Baseline (BL) and 4 weeks post immunisation (4w) timepoints were assayed. The lowest plasma dilution assayed was 1:10, with samples not achieving inhibition at this dilution shown as ‘< 10’. The horizontal black line represents the median, and error bars equal the interquartile range (IQR). Significance was determined by Wilcoxon signed‐rank test between BL and 4w. **(b)** Frequencies of H1^+^ (A/Victoria/2570/2019(H1N1)), H5^+^ (A/Fujian‐Sanyuan/21099/2017) and H1^+^H5^+^ memory B cells in healthy volunteers (*N* = 19) at BL, 1‐ and 4 weeks post‐vaccination (1w and 4w). Donor responses are each timepoint are linked by lines. Significance was determined by the Kruskal–Wallis *H*‐test with Dunn's post‐test comparing each post‐immunisation timepoint to baseline (*P*‐values corrected for multiple comparisons).

Highly cross‐reactive serum antibody responses that bind avian influenza strains are reported to be poorly elicited by inactivated seasonal influenza vaccines.^17^ However, the extent to which cross‐reactive memory B cells are directly elicited by seasonal vaccines remains unclear. Given the recent spread of avian clade 2.3.4.4b H5Nx virus, we sought to characterise cross‐reactive humoral responses to this H5 clade following administration of the 2017 Southern Hemisphere inactivated quadrivalent influenza vaccine (IIV4). To assess whether these responses changed following vaccination, *N* = 23 donors were vaccinated, and we compared their responses at baseline and at 7 days and 1 month post‐immunisation. The HAI assay employed virus particles to measure H1 responses, whereas H5 HA‐ferritin nanoparticles were used as a surrogate for virus to assess H5 responses. As expected, serum HAI titres against A/Michigan/45/2015 pdm09 (H1N1 vaccine component strain) significantly increased 1 month after immunisation (Figure 3a). However, no measurable HAI titres were detected against a clade 2.3.4.4b H5 (A/Fujian‐Sanyuan/21099/2017) prior to or after seasonal vaccination.

The frequency of class‐switched H1^+^, H5^+^ and H1^+^H5^+^ B cells was assessed in individuals (*N* = 23) using B‐cell probes derived from H1 (A/Victoria/2570/2019) and H5 (A/Fujian‐Sanyuan/21099/2017). H1^+^ responses significantly increased at 1 month post‐immunisation (*P* = 0.0431) although the magnitude of the change in frequency was modest (median 1.3‐fold relative to baseline) (Figure 3b). No significant changes in H5^+^ or H1^+^H5^+^ responses were observed up to 1 month post‐vaccination, with only three individuals showing a notable increase in H5‐binding cells post‐vaccination. The distribution of antibody isotypes (Supplementary figure 2a) and B‐cell subsets (as defined by CD21 and CD27 expression) (Supplementary figure 2b) of H1 and H5 reactive B cells were broadly similar to the total memory B‐cell population. Comparison of participant age at the time of vaccination and frequencies of H1 and/or H5 reactive B cells at 4 weeks post‐immunisation did not show any correlation (Supplementary figure 2d). To explore the basis of measurable frequencies of class‐switched H5^+^ B cells in these individuals given they were not expected to have been exposed to H5 virus, we assayed for plasma antibody reactivity against a stabilised HA stem based on the A/Puerto Rico/8/1934(H1N1) (PR8) HA. Titres increased significantly (*P* < 0.0001) 1 week following vaccination before decreasing slightly (but still significantly above baseline) at 1 month after vaccination (Supplementary figure 2c). Moreover, the three individuals that showed the greatest increase in their H5‐binding cell frequency post‐vaccination demonstrated the highest HA stem titres 1 week following immunisation. These data suggest the B cells reactive against the H5 probe are likely targeting conserved regions within the H1 stem, which is representative of Group 1 HAs.

### CD4^+^ T‐cell responses against avian H5 are expanded by seasonal vaccination

**Figure 4:**
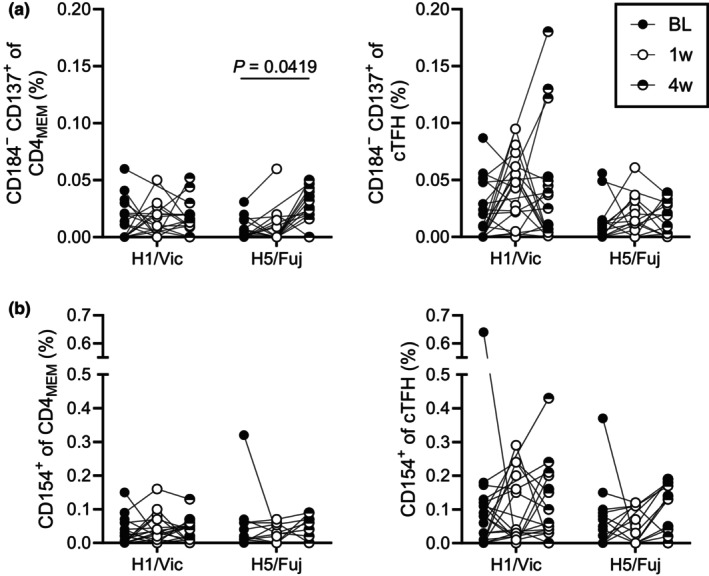
T‐cell responses against H1 and H5 hemagglutinin (HA) following seasonal vaccination. Peripheral blood mononuclear cells (PBMCs) collected from healthy volunteers (*N* = 23) at baseline (BL; black circle) and following seasonal vaccination at 1 week (1w; open circle) and 4 weeks (4w; half‐filled circle) post‐immunisation were tested via AIM assay against H1/Vic [A/Victoria/2570/2019(H1N1)] and H5/Fuj [A/Fujian‐Sanyuan/21099/2017(H5N1)] HA protein. Antigen‐specific CD4^+^ memory T cells (CD4_MEM_) and circulating T follicular helper cells (cTFH) were identified via **(a)** CD184^−^CD137^+^ or **(b)** CD154^+^ gating. Individual donor responses are plotted and linked by lines between timepoints. Significance was determined by the Kruskal–Wallis *H*‐test with Dunn's post‐test comparing each post‐immunisation timepoint to baseline (*P*‐values corrected for multiple comparisons).

CD4^+^ T‐cell responses towards H1 and H5 were assessed via *ex vivo* restimulation and activation‐induced marker (AIM) assay. Given the H5 antigen was expected to be an unencountered antigen for cohort individuals, we employed an H1 antigen [A/Victoria/2570/2019(H1N1)] from a virus isolated after donor samples were collected (in 2017) so that similarly cross‐reactive H1 responses could also be observed. Vaccine‐induced expansion of H1‐specific CD4^+^ cells was primarily observed in the circulating T follicular helper cells (cTFH; CD45RA^−^CXCR5^+^) compartment (Supplementary figure 3), with a 4.6‐fold increase in median CD184^−^CD137^+^ frequency^51^ between baseline and week 1 (*P* = 0.0632), before returning to baseline (Figure 4a). Limited responsiveness was observed within CD4^+^ memory T cells (CD4_MEM_; CXCR5^−^ and not CCR7^+^CD45RA^+^) cells based upon either CD184^−^CD137^+^ or CD154 expression (Figure 4b). H5‐specific CD184^−^CD137^+^ CD4_MEM_ responses were 3.2‐fold higher between baseline and 4 weeks post‐vaccination (*P* = 0.0419). However, there were no significant changes in cTFH responses following IIV4 immunisation.

## Discussion

The rapid global spread of pathogenic avian strains, such as H5Nx 2.3.4.4b, has highlighted the omnipresent threat of an influenza pandemic. Highly cross‐reactive T cell^27^, ^28^, ^29^, ^30^, ^31^ and B cell^14^, ^15^, ^16^ populations have been described in influenza‐exposed adults and may be both a source of background immune protection in the face of a pandemic, and a potential target for expansion via vaccination to broaden immune protection. Here, we show that memory B cells with cross‐reactive recognition of some avian H5 and H7 influenza viruses are detectable in unexposed Australian adults and express immunoglobulins capable of heterosubtypic HA recognition, some with a degree of neutralising activity. As expected and consistent with prior reports, B cells with broadly cross‐reactive recognition of influenza A likely target the HA stem region and are generally drawn from well characterised stereotypic classes (e.g. VH6‐1,^26^, ^49^ VH1‐69,^52^, ^53^, ^54^ VH1‐18^26^). Indeed, seasonal vaccination was observed to boost H1 HA stem‐specific responses that likely explain the appearance of H5 cross‐reactive B cells in three of 23 donors, given H1 and H5 are both Group 1 HAs that have conserved stem regions.^55^ At high concentrations, such antibodies have shown an ability to protect against pathogenic infection in pre‐clinical challenge settings,^17^, ^56^, ^57^, ^58^ including against H5Nx clade 2.3.4.4b.^59^

The protective potential against avian influenza offered by routine seasonal vaccination appears relatively limited, based upon low titres of serum antibody recognising HA from avian strains, no baseline HAI activity and low frequencies of HA‐specific B and T cells. Though, some individuals show evidence of limited cross‐reactive immunity to these unencountered HA antigens, confirming previous reports of limited baseline immunity at a population level.^60^, ^61^, ^62^, ^63^ Upon administration of IIV4, we observed transient boosts in the frequency of H1‐specific B and CD4^+^ T cells as expected. However, this was not recapitulated with regard to H5‐specific responses, where expansion of H5 2.3.4.4b responses was limited to only three of 23 donors. It should be noted that the H5 probe was based on the WHO candidate vaccine virus (CVV) A/Fujian‐Sanyuan/21099/2017(H5N6) belonging to clade 2.3.4.4b rather than a virus from the current outbreak. However, antigenic characterisation via HAI assays has demonstrated that this H5N6 virus and other clade 2.3.4.4b CVVs show similar patterns of antigenic reactivity to clade 2.3.4.4b virus isolates from the outbreak collected from the United States and Canada.^64^ Therefore, screening antibody responses with a clade 2.3.4.4b CCV is expected to provide information relevant to outbreak virus antigenicity. Another factor that may affect measurements of HA‐reactive B‐cell frequency is due to differences in N‐glycosylation occupancy and structure between vaccine‐derived HA and recombinant HA probes. Different cell types have been reported to yield quantitative and qualitative differences in H5N1 HA N‐glycosylation patterns.^65^ At least in the case of donors assayed following vaccination with inactivated virus produced in eggs, the HEK cell line‐produced HA probes likely bear different glycosylation profiles that could influence the ability of certain B‐cell antibodies to bind if their epitope overlaps or incorporates a glycan site.

Similar to the B‐cell responses, our data demonstrate negligible frequencies of H5 clade 2.3.4.4b HA‐specific CD4^+^ memory at baseline in a healthy adult cohort, with only minimal augmentation by seasonal vaccination. T‐cell cross‐reactivity between seasonal and avian influenza strains is likely to be greater for conserved internal proteins, such as nucleoprotein (NP) or matrix protein M1 than for HA, which may constitute a degree of baseline protection in the event of an outbreak. HA‐based vaccines (whether split, recombinant protein or mRNA/LNP), however, rely entirely on T‐cell help derived from the HA antigen. Our data and others suggest the pool of cross‐reactive CD4^+^ T cells established by seasonal influenza exposure is limited, and that pre‐priming is required to support the immunogenicity of H5 vaccines.^33^ Recently, covalent linkage of H5 HA to seasonal HA proteins successfully augmented H5 antibody responses in human tonsil organoids by ‘borrowing’ existing CD4^+^ T‐cell memory.^36^ Establishment of broad population immunity against pre‐pandemic H5Nx strains may thus require multiple vaccine doses to establish a sufficient pool of T‐cell help, or rational design of novel vaccines that maximise availability of pre‐existing CD4^+^ T‐cell immunity.

This study focused on humoral and cellular immunity against HA; however, it is known that antibodies specific for endemic human H1N1 neuraminidase (NA) can afford partial protection against H5N1 virus in animal challenge models given both NAs are of the same serotype.^66^ Recent studies have shown that such NA antibodies are present in human populations and are cross‐reactive with the current avian H5N1 clade 2.3.4.4b NA.^67^, ^68^ Additionally, in animal models, prior infection with the 2009 pandemic H1N1 virus yields protection from severe disease following challenge with clade 2.3.4.4b H5N1 virus from the current outbreak and is associated with H1 HA stem and NA cross‐reactive antibodies.^16^, ^69^, ^70^, ^71^ While the current avian influenza outbreak viruses predominantly utilised N1, previous outbreaks have employed NA subtypes beyond the human endemic N1 and N2;^72^ therefore, avian‐specific vaccine products may benefit from inclusion of HA and NA subtypes that are not already well vaccinated against in human populations. Nonetheless, a limitation of the study presented here is the lack of investigation of NA‐specific responses at baseline and following seasonal influenza vaccination.

Overall, our findings highlight the need for avian influenza‐specific vaccine products to bolster immunity in human populations. Given avian‐origin influenza does not circulate in human populations outside of epidemic outbreaks, it is unlikely that antibody responses elicited by avian virus‐specific human vaccines will affect antigenic drift occurring within avian populations, as can occur with viruses circulating through vaccine‐exposed human populations.^73^ However, prolonged drift within avian populations would likely necessitate vaccine updates. Nonetheless, vaccines targeting avian influenza strains with pandemic potential have been developed and are immunogenic in humans.^7^, ^22^, ^74^ However, better pre‐pandemic preparedness might necessitate consideration of prophylactic immunisation of human populations prior to an outbreak to expand baseline frequencies of H5‐ and H7‐specific memory T and B lymphocytes, albeit with the understanding that strain matching to the strain that facilitates sustained human‐to‐human transmission might be imperfect.

## Methods

### Participant recruitment and sample collection

Study protocols were approved by the University of Melbourne Human Research Ethics Committee (Projects 432/14 and 11395), and all associated procedures were carried out in accordance with the approved guidelines. All participants provided written informed consent in accordance with the Declaration of Helsinki. Participants were not compensated for their participation.

Peripheral blood samples were collected at baseline from a cohort of 18 healthy adults. A group of 23 healthy individuals was administered the 2017 Southern Hemisphere Afluria Quadrivalent (Seqirus) vaccine (IIV4) containing A/Michigan/45/2015(H1N1)pdm09‐like virus, A/Hong Kong/4801/2014(H3N2)‐like virus, B/Phuket/3073/2013‐like virus and B/Brisbane/60/2008‐like virus components. The 2017 IIV4 trial participants provided samples at baseline, 1 week and 4 weeks after immunisation. For all samples, whole blood was collected in sodium heparin anticoagulant. Plasma was collected and stored at −80°C. Peripheral blood mononuclear cells (PBMCs) were collected by Ficoll‐Paque separation, washed and cryopreserved in 10% DMSO/90% fetal calf serum (FCS). PBMCs were stored in liquid nitrogen until use.

### HA‐specific probes and flow cytometry

The design and purification of fluorescently labelled recombinant HA probes with ablated sialic acid binding activity has been previously described.^47^ HA‐specific B cells were identified within cryopreserved PBMC samples by co‐staining with relevant combinations of: H7 [A/Shanghai/01/2013(H7N9)], H1 [A/California/04/2009(H1N1)], H1 [A/New Caledonia/20/1999(H1N1)], H3 [A/Hong Kong/1/1968(H3N2)], H3 [A/Victoria/361/2011(H3N2)] and H5 [A/Indonesia/05/2005(H5N1)] probes conjugated to streptavidin‐PE or streptavidin‐APC (Thermo Fisher Scientific, Waltham, USA), respectively. B cells were characterised using the following: CD3‐QD655 (OKT3, dilution 1:400), CD14‐QD800 (M5E2, dilution 1:200), CD27‐QD605 (O323, dilution 1:100) (Thermo Fisher), CD19‐ECD (J3‐119, dilution 1:100, Beckman Coulter, Brea, USA), IgM‐Cy5.5‐PerCP (G20‐127, dilution 1:100) and IgG‐FITC (G18‐145, dilution 1:50) (BD Biosciences, Franklin Lakes, USA).

For the seasonal influenza vaccine cohort, B cells were co‐stained with H1 [A/Victoria/2570/2019(H1N1)] and H5 [A/Fujian‐Sanyuan/21099/2017(H5N6)] probes conjugated to streptavidin‐APC or streptavidin‐PE, respectively. The staining panel included IgM BUV395 (G20‐127, dilution 1:100), CD21 BUV737 (B‐ly4, dilution 1:100), IgG BV786 (G18‐145, dilution 1:50), IgD PE‐Cy7 (IA6‐2, dilution 1:333) (BD), CD27 BV605 (O323, dilution 1:100; BioLegend, San Diego, USA), CD19 ECD along with BV510 dump makers (CD14, M5E2, dilution 1:200; CD3, OKT3, dilution 1:400; CD8α, RPA‐T8, dilution 1:1000; CD16, 3G8, dilution 1:333; CD10, HI10a, dilution 1:500; all from BioLegend) and unconjugated streptavidin‐BV510 (dilution, 1:400, BD). For all samples, cell viability was assessed using Aqua Live/Dead amine‐reactive dye (Thermo Fisher). One to two million events were collected on an LSR II instrument (BD) or a FACSymphony A5 SE (BD). Analysis was performed using the FlowJo software version 9.5.2 or 10.10 (BD).

### Activation‐induced marker assay

Cryopreserved PBMC samples were thawed and rested for 2–4 h at 37°C in RPMI‐1640 supplemented with penicillin/streptomycin/L‐glutamate and 10% FCS (Merck, Darmstadt, Germany) (RF10). Cells were cultured at 1–2 million cells per well in 200 μL in 96‐well plates (Corning, Corning, USA) and stimulated for 20 h with 5 μg mL^−1^ of protein [BSA, A/Victoria/2570/2019(H1N1) HA or A/Fujian‐Sanyuan/21099/2017(H5N6) HA]. Small pools of selected donors were also stimulated with SEB (5 μg mL^−1^) as a positive control. CD154 PE (TRAP1, dilution 1:100, BD) was included in the medium during stimulation. Following stimulation, cells were washed and stained with monoclonal antibodies (mAbs) CD183 PE‐Dazzle594 (G02H57, dilution 1:100, BioLegend), CD184 BUV395 (12G5, dilution 1:200, BD) and CD185 PE‐Cy7 (MU5UBEE, dilution 1:66.7, Thermo Fisher) for 30 min at 37°C. Cells were then washed, stained with Live/Dead Aqua viability dye (Thermo Fisher) and incubated with mAbs CD3 BUV805 (SK7, dilution 1:200), CD20 BV510 (2H7, dilution 1:200), CD45RA R718 (HI100, dilution 1:100) (BD), CD137 BV421 (4B4‐1, dilution 1:200), CD14 BV510 (M5E2, dilution 1:200), CD4 BV605 (RPA‐T4, dilution 1:200), CD196 BV785 (G034E3, dilution 1:200), CD134 PerCP‐Cy5.5 (ACT35, dilution 1:100) and CD197 Ax647 (G043H7, dilution 1:50) (BioLegend) for 30 min at 4°C. Cells were washed, fixed with 1% formaldehyde and acquired on a BD FACS Symphony A5 SE and analysis was performed using the FlowJo Software v10.10.

### Sequencing, cloning and expression of B‐cell immunoglobulins

The sequencing and cloning of BCRs from single B cells was performed as previously described.^48^, ^75^ Plasmids expressing heavy and light immunoglobulin chains were transfected into Expi293F cells using ExpiFectamine (Thermo Fisher). Recombinant monoclonal antibodies were purified from culture supernatants using Protein‐A or G (Thermo Fisher) as per the manufacturer's instructions.

### Enzyme‐linked immunosorbent assay

Antibody binding to HA was tested by enzyme‐linked immunosorbent assay (ELISA). 96‐well Immunosorp plates (Thermo Fisher) were coated overnight at 4°C with 2 μg mL^−1^ recombinant HA either expressed in‐house in Expi293 cells as previously described^47^ or sourced commercially (Sino Biological, Beijing, China). Assays measuring antibody binding to HA stem used identical ELISA plate coating conditions with recombinant stabilised HA stem protein engineered for A/Puerto Rico/8/1934(H1N1) (PR8) as previously described.^76^, ^77^ After blocking with 1% FCS in PBS, duplicate wells of monoclonal antibodies (starting at 10 μg mL^−1^, fourfold serial dilutions) or human sera (1:100, four‐fold serial dilutions) were added and incubated for 1 h at room temperature. Plates were washed prior to incubation with a 1:20 000 dilution of HRP‐conjugated anti‐human IgG (LGC Clinical Diagnostics, Milford, USA) for 1 h at room temperature. Plates were washed and developed using 3,3′,5,5′‐Tetramethylbenzidine (TMB) substrate and read at 450 nm. HA‐binding activity of monoclonal antibodies was calculated as the antibody concentration giving half‐maximal signal (EC50) using a fitted curve (4 parameter log regression). For serum samples, endpoint titres were determined using a fitted curve (4 parameter log regression) and a cut‐off of two times background.

### HAI assay

Serum HAI was performed according to the WHO Global Influenza Surveillance Network protocols^78^ with the exception that assays for H5 HA utilised H5‐ferritin nanoparticles instead of the virus (described below). Samples were treated with receptor‐destroying enzyme (Denka Sieken, Tokyo, Japan) at a 1:3 ratio for 18 h at 37°C, heat inactivated at 56°C for 30 min and adsorbed with 5% erythrocytes before testing. The A/Michigan/45/2015 (H1N1) virus was propagated in Day‐10 embryonated chicken eggs. For H5‐ferritin nanoparticles, genes expressing the ectodomain of H5 A/Fujian‐Sanyuan/21099/2017 HA were synthesised (IDT, Coralville, USA) and cloned into a mammalian expression vector allowing the expression of ferritin nanoparticles as described previously.^79^ H5‐ferritin nanoparticles were expressed using Expi293F cells (Thermo Fisher) and purified using HiTrap Anion exchange and CaptoCore chromatography (Cytiva, Marlborough, USA). Purified nanoparticles were diluted to 4 HA units for use in HAI assays.

Assessment of HAI activity of recombinant mAbs was assessed using 1% turkey erythrocytes in a WHO standardised assay. Briefly, mAbs were diluted to 100 μg mL^−1^ in PBS prior to incubation with influenza viruses from strains A/California/07/2009, A/Indonesia/5/2005/PR8‐IBDC‐RG2, NIBRG‐268 A/Anhui/1/2013, A/Hong Kong/4801/2014, B/Phuket/3073/2013 and B/Brisbane/60/2008. HAI titres are reported as the reciprocal of the highest dilution where hemagglutination was completely inhibited.

### FRA assay

Neutralisation activity of recombinant mAbs against A/California/07/2009, A/Indonesia/5/2005/PR8‐IBDC‐RG2, NIBRG‐268 A/Anhui/1/2013, A/Hong Kong/4801/2014, B/Phuket/3073/2013 and B/Brisbane/60/2008 was examined using FRAs as previously described.^80^ The neutralisation titre is expressed as the reciprocal of the highest dilution of a 1 mg mL^−1^ mAb stock at which virus infection is inhibited by ≥ 50%.

### Statistical analyses

Data are generally presented as median ± interquartile range (IQR). Statistical analysis typically employed non‐parametric tests. Specifically, the Wilcoxon signed‐rank test was used for comparisons of two groups, and the Kruskal–Wallis *H*‐test with Dunn's post‐test for comparisons of three or more groups. Some analyses of datasets with matched samples utilised mixed‐effects ANOVA analysis, with the Geisser–Greenhouse correction (to accommodate any missing timepoint samples), followed by Tukey's multiple comparisons test. Datasets were assessed for normality using both the D'Agostino‐Pearson ‘omnibus K2’ and Shapiro–Wilk tests. Correlations were assessed using Spearman's Rank Correlation. All statistical analyses were performed using GraphPad Prism v5.0 or v10.4.2 (Dotmatics, Boston, USA).

## Author contributions

CAG, JAJ and AKW designed the study and experiments. CAG, MK, RE, MZMZ, Y‐CL, AKZ, LSUS and ACH performed experiments. SJK provided unique samples. CAG, MK, JAJ and AKW analysed the experimental data. CAG, JAJ and AKW wrote the original manuscript draft. All authors reviewed, edited and approved the manuscript.

## Conflict of interest

The authors declare no conflict of interest.

## Supporting information


Supplementary figure 1–3

## Data Availability

The data that support the findings of this study are available from the corresponding author upon reasonable request.
