# Supplementary material for: Low‐level human memory T and B cells recognising avian influenza hemagglutinins are poorly responsive to existing seasonal influenza vaccines
Source: Clin Transl Immunology. 2025 Dec 10;14(12):e70067. doi: 10.1002/cti2.70067 (PMC12695479; doi:10.1002/cti2.70067)
Supplement: Supplementary file 1 — Supplementary figure 1–3 [file CTI2-14-e70067-s001.docx]

# **Supporting information**

# Title: Low-level human memory T and B cells recognizing avian influenza hemagglutinins are poorly responsive to existing seasonal influenza vaccines

Running Title: Avian HA response to seasonal flu vaccines

Authors: Christopher A Gonelli^1^, Marios Koutsakos^1^, Robyn Esterbauer^1^, Ming ZM Zheng^1^, Yee-Chen Liu^1^, Amanada Kyaw Zin^1^, Lara S U Schwab^1^, Aeron C Hurt^2^, Stephen J Kent^1^, Jennifer A Juno^1^, Adam K Wheatley^1^

Affiliations: ^1^Department of Microbiology and Immunology, The University of Melbourne at the Peter Doherty Institute for Infection and Immunity, Melbourne, VIC, Australia; ^2^WHO Collaborating Centre for Reference and Research on Influenza, Peter Doherty Institute for Infection and Immunity, Melbourne, VIC, Australia.

*Address correspondence to: Adam K Wheatley (a.wheatley@unimelb.edu.au)

**
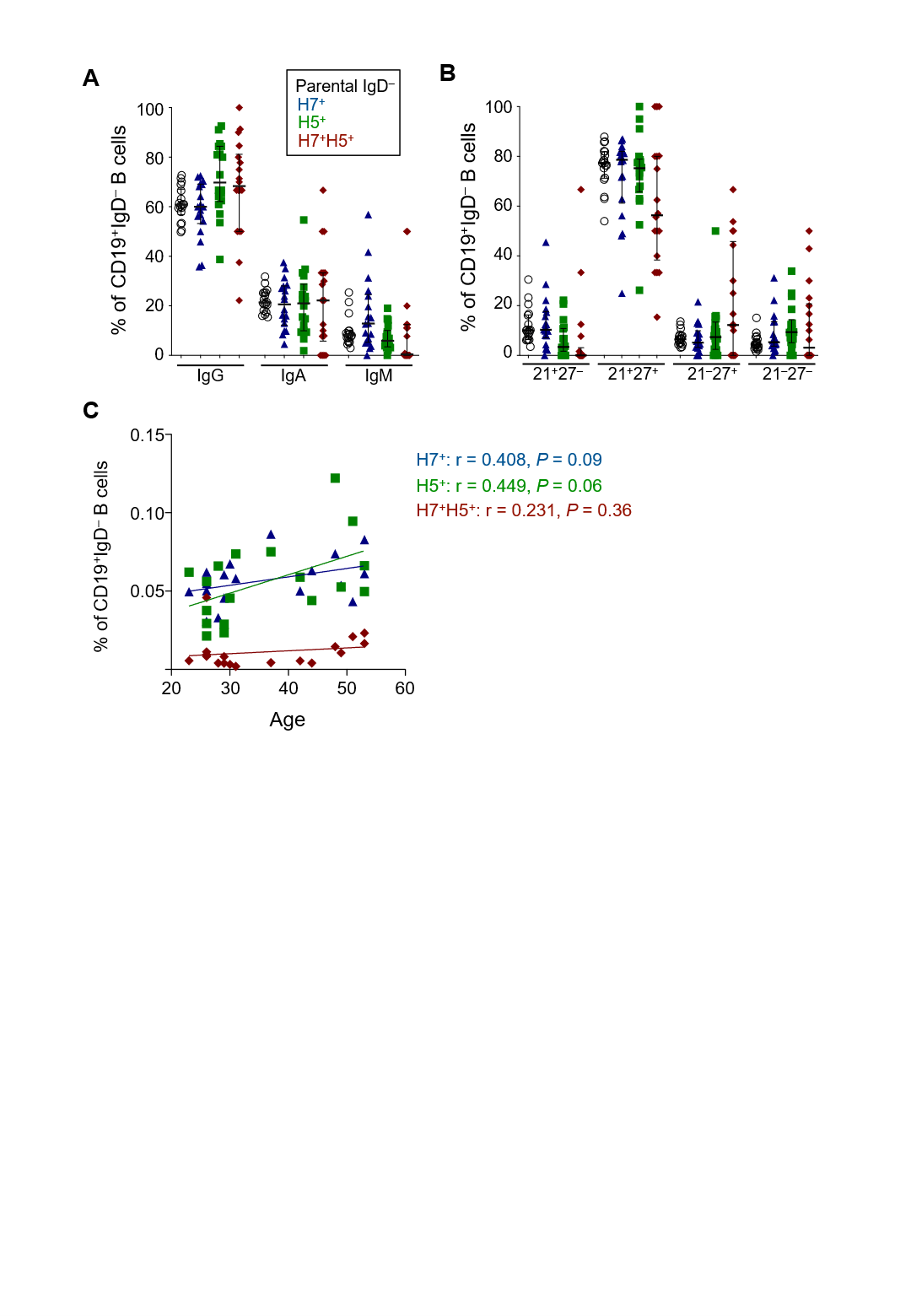
**

**Supplementary figure 1: Subclass, phenotype and age correlation of H7 and H5 HA-specific memory B cells.
(a)** Distribution of IgG, IgA and IgM expression on H7^+^ (blue), H5^+^ (green), H7^+^H5^+^ (red) and the parental memory B cell population (open circles) in healthy volunteers (N = 18). **(b)** Distribution of B cell subsets delineated by CD27 and CD21 staining: CD27^–^CD21^+^ naïve, CD27^+^CD21^+^ resting memory, CD27^+^CD21^–^ activated memory and CD27^–^CD21^–^ tissue-like populations. Lines indicate median and IQR. **(c)** Correlation between participant age and HA-specific memory B cell frequencies (N = 18). Significant differences were assessed by the Spearman’s Rank Correlation.

**
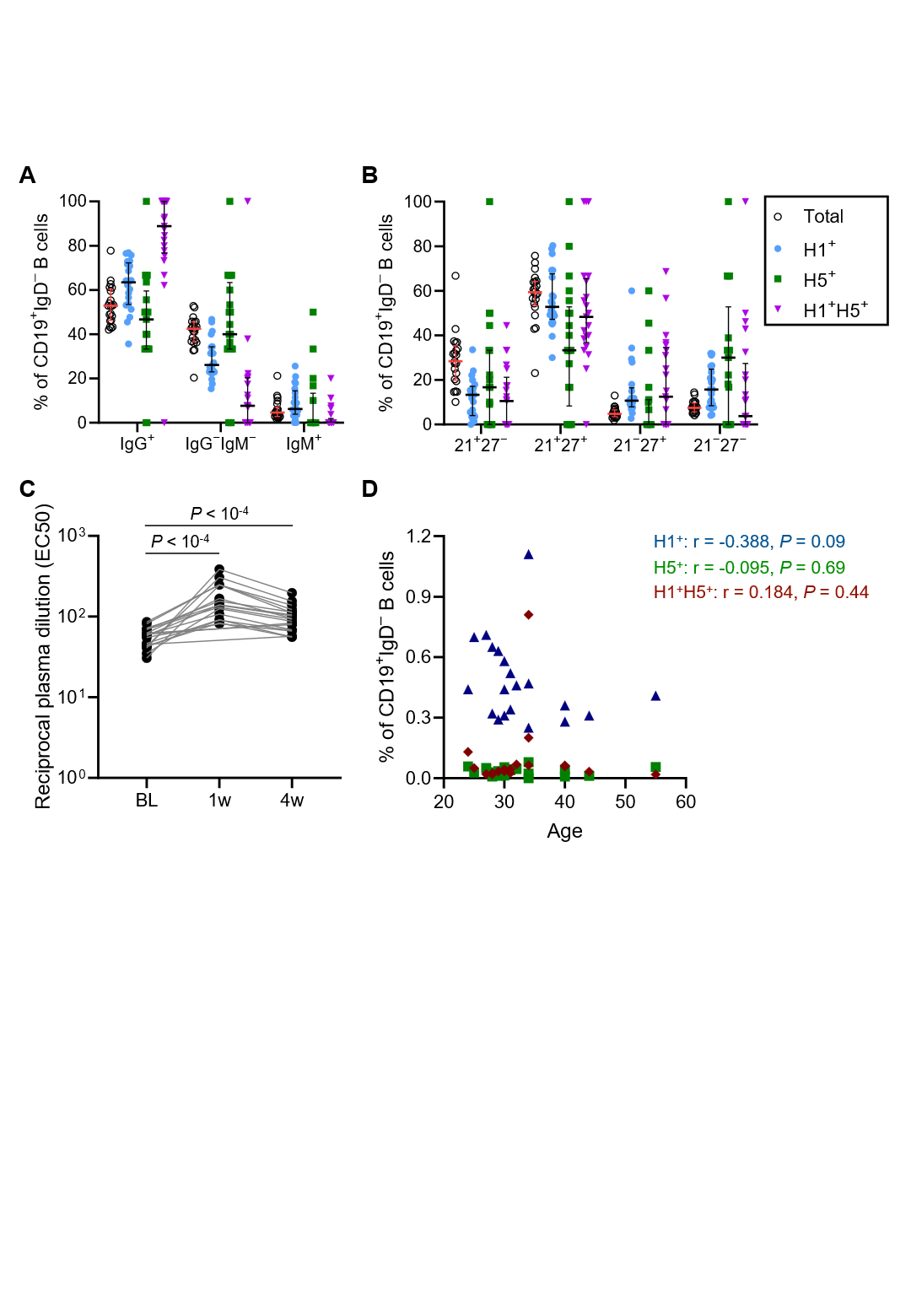
**

**Supplementary figure 2: Subclass, phenotype and age correlation of H1 and H5 HA-specific memory B cells and HA stem-specific plasma antibody responses.
(a)** Distribution of IgG, IgG^–^IgM^–^ and IgM expression on H1^+^ (light blue), H5^+^ (green), H1^+^H5^+^ (purple) and the parental memory B cell population (total IgD^–^, open circles) in 2017 IIV4 vaccine cohort participants (N = 23) at 4 weeks post immunization. **(b)** Distribution of B cell subsets delineated by CD27 and CD21 staining: CD27^–^CD21^+^ naïve, CD27^+^CD21^+^ resting memory, CD27^+^CD21^–^ activated memory and CD27^–^CD21^–^ tissue-like populations. **(a, b)** Horizontal black lines indicate the median and error bars equal the IQR (lines colored red for “total IgD^–^” for clarity). **(c)** A subset of participant plasma samples from the 2017 IIV4 vaccine cohort (N = 20) were assayed for reactivity against PR8 HA stabilized stem antigen at baseline (BL), 1 week (1w) and 4 weeks (4w) post immunization. Reciprocal plasma dilutions yielding half maximal binding (EC50) are shown with lines linking participant samples. To determine significance, mixed-effects ANOVA analysis, with the Geisser-Greenhouse correction, was used on matched samples followed by Tukey’s multiple comparisons test on all pairs of timepoints. The post-test adjusted *P* values are shown on the graph. **(d)** Correlation between participant age and HA-specific memory B cell frequencies (N = 23) at 4 weeks post-immunization. Significant differences were assessed by the Spearman’s Rank Correlation.

**
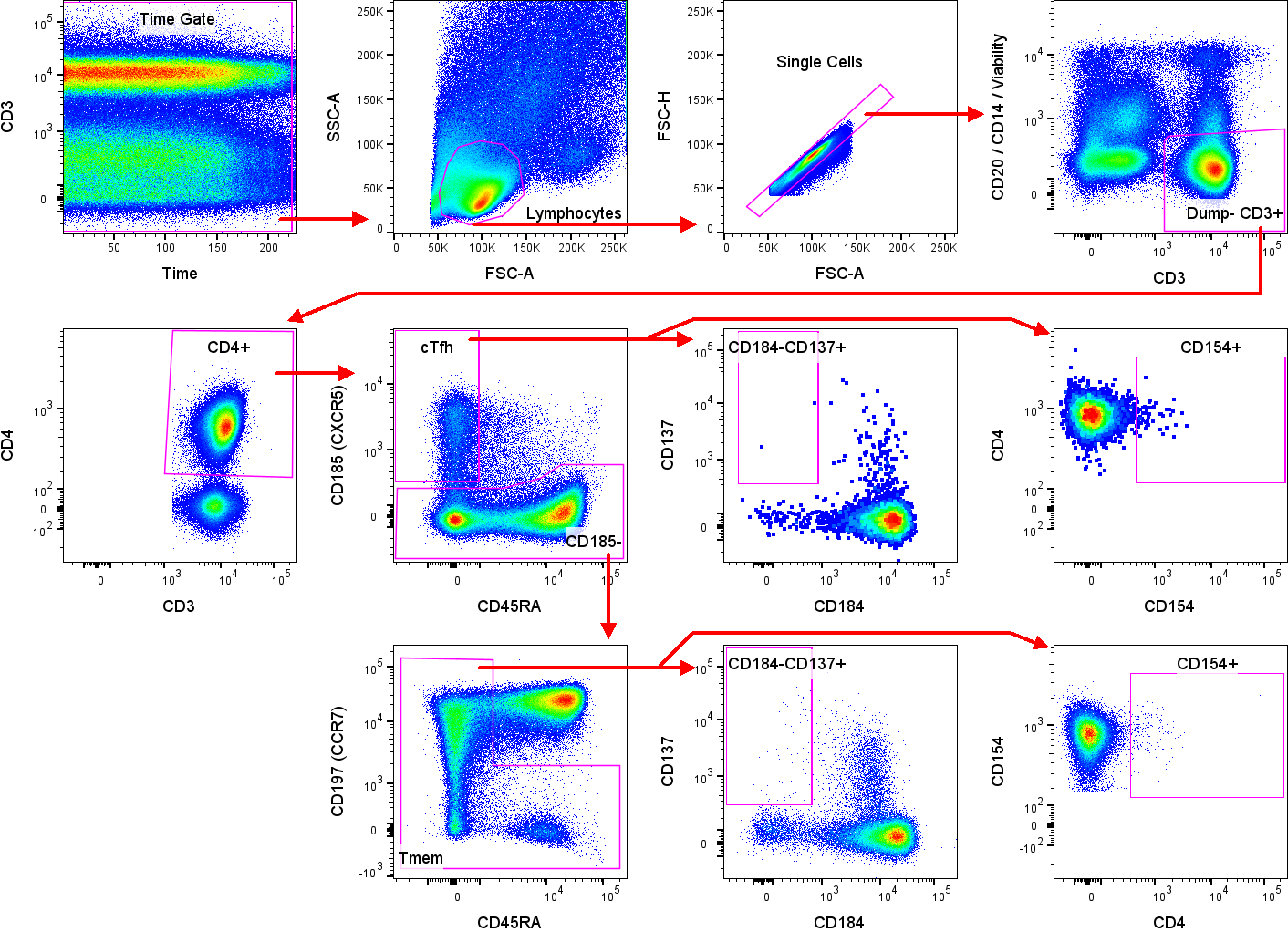
**

**Supplementary figure 3: Flow cytometry gating for AIM assay.**
Staining of cryopreserved PBMCs to identify circulating T follicular helper (cTFH) cells (CXCR5^+^) and memory T (Tmem) cells (CXCR5^−^ and not CCR7^+^CD45RA^+^). For each T cell subset, antigen specific cells were identified by CD137^+^CD184^−^ or CD154^+^ staining.
